# Clinical and Radiographic Evaluation of Biodentine for Apexogenesis in Young Permanent Mandibular Molars: A Systematic Review and Meta-Analysis

**DOI:** 10.7759/cureus.88240

**Published:** 2025-07-18

**Authors:** Sujatha Paranna, Rasika Gogawale, Anil Patil, Sandhyarani Huddar, Kiran Patro, Janhavi Kanetkar

**Affiliations:** 1 Pedodontics and Preventive Dentistry, Bharati Vidyapeeth (Deemed to be University) Dental College and Hospital, Sangli, IND

**Keywords:** apexogenesis, biodentine, carious lesions, mta, permanent mandibular molars

## Abstract

Apexogenesis, a vital pulp therapy, is a crucial treatment option for preserving the vitality of immature permanent teeth affected by caries or trauma. The goal of apexogenesis is to promote the continued development of the root and apex, allowing for a more favorable prognosis. This systematic review and meta-analysis aimed to evaluate the clinical and radiographic outcomes of Biodentine (Septodont, Saint-Maur-des-Fossés, France) for apexogenesis in young permanent mandibular molars. The protocol of the present article was registered in the PROSPERO database and allocated the identification number CRD42024589204. A comprehensive search of PubMed, Scopus, and EMBASE was conducted using a combination of Boolean operators (AND, OR) and Medical Subject Headings (MeSH) keywords to retrieve relevant studies in accordance with the Preferred Reporting Items for Systematic Reviews and Meta-Analyses (PRISMA) protocol. The formulated population, intervention, comparison, and outcome (PICO) question was "What are the clinical and radiographic outcomes for apexogenesis in young permanent mandibular molars?" Six trials were considered for inclusion, with the studies demonstrating that Biodentine could be used in stepwise root canal retreatment with/without coronal seal and indirect pulp treatment. However, the studies also highlighted the importance of individual clinical circumstances and preference in choosing the treatment material. The meta-analysis revealed that Biodentine tends to present slightly higher clinical success rates compared to other calcium compounds, although the difference was not statistically significant. The heterogeneity test revealed moderate variability between studies. Regarding radiographic success rates, the analysis showed that Biodentine tends to have similar radiographic success rates compared to mineral trioxide aggregate (MTA) at six-month and one-year follow-up periods. The overall summary estimate revealed no significant difference between Biodentine and MTA. The heterogeneity test did not detect significant variability between studies at both follow-up periods. Our systematic review suggests that Biodentine has similar clinical and radiographic success rates compared to other calcium compounds and MTA for apexogenesis in young permanent mandibular molars. However, the results should be interpreted with caution due to the moderate heterogeneity and the limited number of studies included.

## Introduction and background

In recent times, a new generation of endodontic cements known as calcium silicate cements has emerged as a possible alternative to calcium hydroxide (CH) in critical pulp therapy [1-3]. Among these, Biodentine, developed by Septodont, Saint-Maur-des-Fossés, France, has been the most featured product because of its exceptional physical, chemical, and biological qualities. Biodentine is a tricalcium silicate cement with exceptional biocompatibility and bioactivity, along with outstanding sealing ability. In contrast to conventional calcium silicate cement, it exhibits a faster setting time and higher mechanical strength; for this reason, it could be utilized as a substitute for dentine or as a pulp-capping agent [4,5].

The two-step excavation technique seeks to decrease the danger of pulp exposure and maintain pulp vitality and was introduced to complement the usual technique of total caries removal [6]. The initial phase of this procedure involves partial removal of the carious tissue, leaving over the pulp a layer of damaged dentin. Then, a cavity is provisionally filled with a temporary restoration to encourage the production of reparative dentin. Later, during the second stage, the cavity is reopened, and the remaining demineralized dentin is dug [5]. Although this technology has promised much, it is not without certain disadvantages. The disadvantages, as highlighted, comprise two visits and the higher potential of pulp exposure during the second step [6]. Based on these data, a strategy for partial caries eradication was created in one visit. The one-visit approach demonstrated improved long-term success rates compared to a two-step excavation method in a randomized clinical trial study [7,8]. However, it has equally been documented that partial excavation of caries leads to lower cases of pulp exposure than when the carious process is totally eliminated [9].

There have been various studies done on the clinical and radiological effects of Biodentine in vital pulp therapy in permanent teeth [10,11]. The majority were performed on permanent teeth rather than young permanent ones with immature root apexification or apexogenesis employing Biodentine, whereas just a few investigations have been completed. In this sense, the mandibular first molar is of special relevance because it is one of the first permanent teeth to erupt and is very often involved with deep carious lesions when the root formation is still incomplete [12].

Given the small volume of available evidence and the limited consensus on the best material and technique for vital pulp therapy in young permanent mandibular molars, this systematic review and meta-analysis aimed to critically appraise and summarize the available evidence related to the clinical and radiographic outcomes of Biodentine used for apexogenesis in young permanent mandibular molars.

## Review

Materials and methods

Eligibility Criteria

The protocol of the present article was registered in the PROSPERO database and allocated the identification number CRD42024589204.

**Figure 1:**
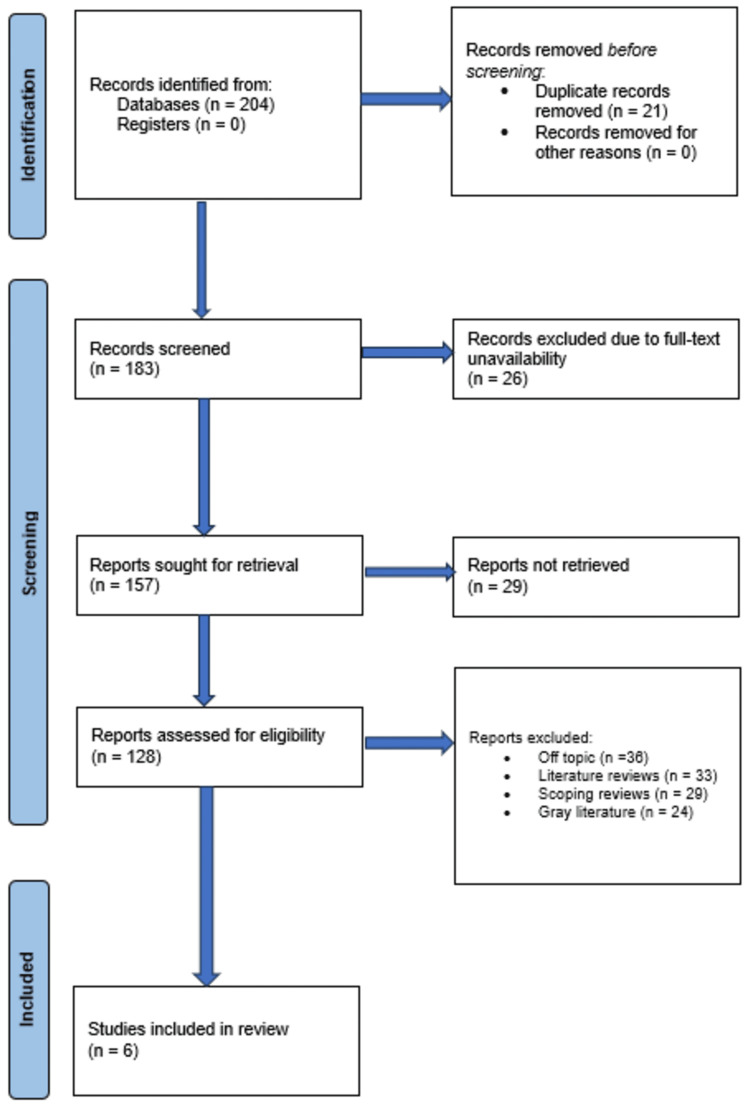
Description of the different stages of the article selection process for the review

Our review was designed using the PICO protocol in addition to following the PRISMA reporting guidelines [13]. The PICO framework consisted of the fundamental elements: population, intervention, comparison, and outcome (Figure 1).

The involved population consists of young permanent mandibular molars with vital pulps in need of pulpotomy. The exposure will be Biodentine being used as the material for pulp capping. The comparator was another pulp capping material, such as mineral trioxide aggregate (MTA) or calcium hydroxide. Clinical and radiographic success were the primary measured outcomes. The selection criteria formulated for the review are displayed in Table 1.

**Table 1:** Inclusion and exclusion criteria devised for this review

Inclusion criteria	Exclusion criteria
Studies using Biodentine as the pulp capping material	Studies involving primary teeth
Studies employing the pulpotomy procedure	Studies using other pulp capping materials
Studies focusing on young immature permanent mandibular molars with vital pulps	Studies including non-vital teeth
Studies providing both clinical and radiographic assessments of treated teeth	
Studies published between January 2000 and June 2024	

Database Search Protocol

PubMed, Scopus, and EMBASE were searched using a combination of Boolean operators (AND, OR) and Medical Subject Headings (MeSH) keywords to maximize the retrieval of relevant studies (Table 2). The search strategy was modified for each database and included population, exposure, comparison, and outcomes of interest. The search included literature from January 2000 to June 2024, written in English. Reference lists from the ascertained articles were also searched to find other appropriate studies.

**Table 2:** Database search strings utilized across the different databases

Database	Search string
PubMed	(("Molar, Permanent"[Mesh]) AND ("Mandible"[Mesh])) AND (("Biodentine"[Mesh]) OR (((tricalcium silicate) AND (cement)) OR (Biodentine))) AND (("Pulpotomy"[Mesh]) OR "Root Canal Therapy"[Mesh]) AND ((("Apexogenesis"[Mesh]) OR "Root Development"[Mesh]) OR (("Radiography"[Mesh]) OR "Clinical Success"))
Scopus	(TITLE-ABS-KEY(("permanent molar*" AND mandib* AND (biodentine OR "tricalcium silicate cement"))) AND TITLE-ABS-KEY(pulpotomy OR "root canal therapy") AND TITLE-ABS-KEY(apexogenesis OR "root development" OR radiograph* OR "clinical success"))
EMBASE	("permanent tooth"/exp OR "permanent tooth" OR "permanent molar*") AND ("mandible"/exp OR mandib*) AND ("biodentine"/exp OR biodentine OR "tricalcium silicate cement") AND ("pulpotomy"/exp OR pulpotomy OR "root canal therapy"/exp) AND ("apexogenesis"/exp OR apexogenesis OR "tooth root development"/exp OR "root development" OR "radiography"/exp OR radiograph* OR "clinical success")

Data Extraction Protocol

A standardized data extraction form was developed and utilized for extracting relevant data from the selected studies. Data extraction was done from the included studies by two independent reviewers (Rasika Gogawale (RG) and Kiran Patro (KP)). Two reviewers did a manual search for papers to potentially include in the review, depending on the references of selected research. Any disagreement that occurred was resolved by a third reviewer (Sujatha Paranna (SP)).

The extracted data items included, but were not limited to, the following characteristics of the study: intervention, pulpotomy technique, pulp capping material used, and restoration type; follow-up duration; and outcome measures in the form of clinical success, radiographic success, apical closure, root development, and adverse events. Whenever necessary, authors of the included studies were contacted to obtain missing or unclear data.

Risk of Bias Assessment Protocol

The Cochrane risk-of-bias tool for randomized trials (RoB 2) version 2 is the recommended tool to assess the risk of bias in randomized trials included in Cochrane Reviews. RoB 2 is structured into a fixed set of domains of bias, focusing on different aspects of trial design, conduct, and reporting.

Cochrane's RoB 2.0 [14] tool was utilized to evaluate the risk of bias in the studies included in this investigation. Two reviewers separately evaluated the risk in each included study, and every dispute was settled by consensus.

Meta-Analysis Protocol

We carried out the meta-analysis using RevMan 5 version 5.4.1 (The Cochrane Collaboration, London, UK). The analysis was performed with the following main outcomes: to estimate the clinical and radiographic success rates of Biodentine and MTA in apexogenesis procedures. The analyses were done using the random effects (RE) model, taking into consideration the expected heterogeneity among studies. The generated forest plots presented the odds ratio (OR) with 95% confidence interval (CI) for every included study and the overall summary estimate.

Results

Article Inclusion Process

A total of 204 documents were initially identified from databases, with very few records discovered in registers. Following the removal of duplicate entries (n = 21), 183 records in all were screened. A total of 157 reports were requested for retrieval due to the exclusion of title, from which 29 entries were removed due to the lack of complete content. Therefore, 128 reports were eventually assessed for eligibility. Following a thorough evaluation, 36 papers were deemed irrelevant to the topic, 33 contained reviews of previous research, 29 were scoping studies, and 24 were labeled as grey literature, which led to their removal. Six studies [15-20] met the qualifying requirements and were included in the study after thorough evaluation.

Assessment of Bias

**Figure 2:**
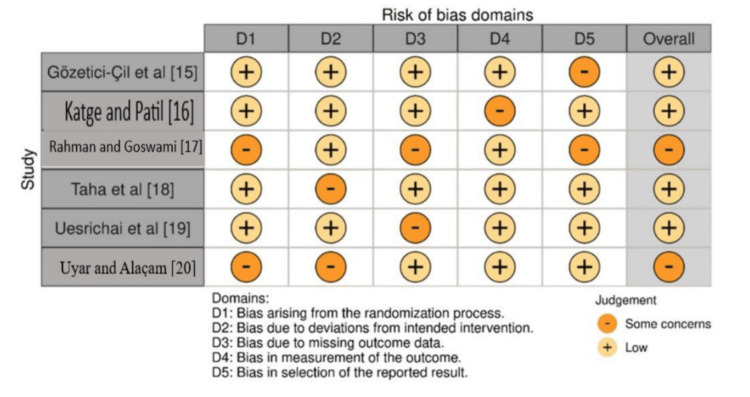
Bias assessment using the RoB 2.0 tool across the included trials

As elucidated in Figure 2, the bias assessment showed that Gözetici-Çil et al. [15] and Katge and Patil [16] had an overall low risk of bias, with most domains rated as low, similar to Taha et al. [18] and Uesrichai et al. [19]. In contrast, Rahman and Goswami [17] and Uyar and Alaçam [20] had an overall risk of bias rated as some concerns, with multiple domains rated as some concerns. Risk of bias domains are categories that help assess potential systematic errors in research studies, ensuring findings are reliable and trustworthy, and include selection, performance, detection, attrition, reporting, and other biases.

Assessment of Baseline Variables

Inferences drawn from the included trials are shown in Table 3 [15-20]. The RCT by Gözetici-Çil et al. involved 165 teeth from patients ranging in age from 13 to 65, with a two-year follow-up period [15]. Katge and Patil conducted a study on 42 molars, focusing on children between the ages of seven and nine; the follow-up period was rather short at 12 months [16]. Forty-nine individuals with a mean age of 12.36 ± 2.29 years were studied by Rahman and Goswami; the study has a 24-month follow-up period [17]. In 2022, 156 teeth were analyzed by Taha et al.; the mean age of the patients was 20 years, and the follow-up period was one year [18]. In 2019, Uesrichai et al. examined 69 teeth from 69 patients, ages ranging from six to 18, over a maximum of six-year follow-up period [19]. Uyar and Alaçam examined 54 teeth from 50 patients, ages 6-13, with a follow-up period of 1-12 months [20].

**Table 3:** Included trials and their observed inferences SRSD: selective removal of soft dentin, CS: calcium silicate, PE: pulp exposure, MTA: mineral trioxide aggregate, GIC: glass ionomer cement, BC RRM: bioceramic root repair material, RCT: randomized controlled trial, RMGI: resin-modified glass ionomer, CH: calcium hydroxide

Study	Year	Sample size	Age range	Follow-up period	Treatment groups	Success rate	Radiographic outcomes	Notable findings	Trial design	Interventions	Overall inference drawn
Gözetici-Çil et al. [15]	2023	165 teeth	13-65 years	2 years	SRSD with/without CS, control	100% (SRSD), 93.5% (SRSD without CS), 82.4% (control)	No significant difference in success rates between groups	Higher postoperative pain in the PE group, significant difference in survival curves between the test and control groups	Double blind	Local anesthesia, removal of carious tissue, restoration with resin composite	SRSD with/without CS is a viable treatment option for deep posterior primary caries lesions, with a high success rate and minimal postoperative pain.
Katge and Patil [16]	2017	42 molars	7-9 years	12 months	Biodentine, MTA	100% (both groups)	95.24% (Biodentine), 85.71% (MTA) (dentin bridge formation)	No statistical difference between Biodentine and MTA in clinical and radiographic parameters	Split-mouth design	Direct pulp capping procedure, local anesthesia, rubber dam isolation, carious lesion removal, Biodentine or MTA application	Biodentine and MTA are both effective in promoting dentin bridge formation and pulp vitality in young permanent molars with carious involvement.
Rahman and Goswami [17]	2021	49	12.36 ± 2.29 years (mean)	24 months	Biodentine, Theracal, Dycal	94.44% (Biodentine), 100% (Theracal), 77.78% (Dycal)	No significant difference in radiographic outcomes between groups	Statistically significant difference in overall success rates between groups at 24 months (p = 0.03)	Double blind, simple randomization	Indirect pulp treatment with Biodentine, Theracal, or Dycal, followed by restoration with GIC and resin composite	Biodentine is a viable option for indirect pulp treatment, with a high success rate and minimal postoperative pain.
Taha et al. [18]	2022	156 teeth	20 years (mean)	1 year	ProRoot MTA, Biodentine, TotalFill BC RRM Fast Set Putty	91.8% (TotalFill), 93.3% (MTA), 91.9% (Biodentine)	Absence of pathosis on recall radiograph, complete radiographic healing	No significant difference in success rates between materials (p > 0.05), significant reduction in pain scores	Single-blinded RCT	Pulpotomy procedure with ProRoot MTA, Biodentine, or TotalFill BC RRM Fast Set Putty, followed by restoration with RMGI liner and direct resin-based composite	Biodentine is an effective pulp capping material for pulpotomy procedures, with a high success rate and significant reduction in pain scores.
Uesrichai et al. [19]	2019	69 teeth (69 patients)	6-18 years	Up to 6 years	ProRoot MTA, Biodentine	90% (ProRoot MTA), 87% (Biodentine)	Continued root formation, improvement of early periapical changes, absence of prominent periapical lesion, and internal and/or external root resorption	Biodentine non-inferior to ProRoot MTA, higher incidence of grey discoloration with ProRoot MTA	RCT	Topical anesthesia, local anesthesia, cavity preparation, carious removal, pulpal tissue removal, sodium hypochlorite irrigation, ProRoot MTA or Biodentine application, protective base placement, resin composite or stainless steel crown restoration	Biodentine suitable alternative to ProRoot MTA for partial pulpotomy in pediatric patients.
Uyar and Alaçam [20]	2021	54 teeth (50 patients)	6-13 years	1-12 months	CH powder, MTA, Biodentine	87% (overall success rate)	Root development continuity, integrity, and/or discontinuity of lamina dura, presence of radiolucency in bifurcation or periapical area, apical closure stages	No statistically significant difference between the three groups, higher clinical success rate with MTA and Biodentine	Prospective clinical study	Complete caries removal, placement of protective liner, direct pulp therapy, partial pulpotomy with biocompatible pulp medicament, glass ionomer cement liner, stainless steel crowns	No significant difference between the three groups; MTA and Biodentine showed higher clinical success rates.

Observation of Treatment

Gözetici-Çil et al. conducted a double-blind study comparing selective removal of soft dentin (SRSD) with or without calcium silicate (CS) cement to a control group [15]. Katge and Patil employed a split-mouth design to evaluate Biodentine and MTA in a direct pulp capping process [16]. Rahman and Goswami adopted a double-blind, basic randomization method to examine Biodentine, Theracal, and Dycal in indirect pulp therapy [17]. Taha et al. conducted a single-blind RCT to compare ProRoot MTA, Biodentine, and TotalFill Bioceramic Root Repair Material (BC RRM) Fast Set Putty in pulpotomy procedures [18]. Uesrichai et al. conducted an RCT to test CH powder, MTA, and Biodentine in pulpotomy procedures [19]. Uyar and Alaçam conducted a prospective clinical trial to investigate the efficacy of direct pulp therapy with biocompatible pulp medicaments [20].

Outcome Observation

Clinical outcome: Gözetici-Çil et al. found success rates of 100% for SRSD, 93.5% for SRSD without CS, and 82.4% for the control group, with no significant difference in success rates between groups [15]. Katge and Patil showed a 100% success rate for both Biodentine and MTA groups, with no statistical difference in clinical characteristics between the two materials [16]. Rahman and Goswami found success rates of 94.44% for Biodentine, 100% for Theracal, and 77.78% for Dycal, with no significant difference in clinical outcomes between groups [17]. Taha et al. showed success rates of 91.8% for TotalFill, 93.3% for MTA, and 91.9% for Biodentine, with no significant difference in success rates between materials and a significant reduction in pain [18].

Radiographic outcome: Katge and Patil showed a 100% success rate for both Biodentine and MTA groups, with no statistical difference in radiographic characteristics between the two materials [16]. Uesrichai et al. reported a success rate of 90% with ProRoot MTA and 87% for Biodentine, with ongoing root development and improvement of early periapical alterations [19]. Uyar and Alaçam found an overall success rate of 87%, with no statistically significant difference between the three groups; MTA and Biodentine demonstrated a higher clinical success rate [20].

Meta-Analytical Findings

**Figure 3:**
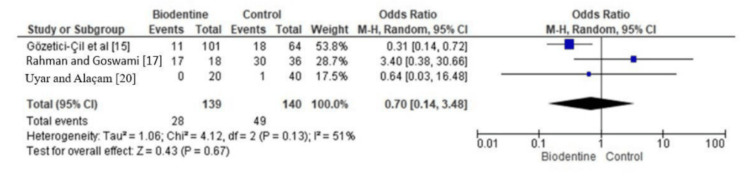
Clinical success rate of Biodentine as compared to other Ca compounds (in terms of pulpal involvement and pain presentation)

The forest plot in Figure 3 shows the clinical success rate in terms of pulpal involvement and presentation of pain of Biodentine with other Ca compounds across three trials [15,17,20]. The summary estimate of the odds ratio (OR) was 0.70, showing a trend for Biodentine to present slightly higher clinical success rates when compared with the other Ca compounds. However, this difference was not statistically significant, with moderate heterogeneity.

**Figure 4:**
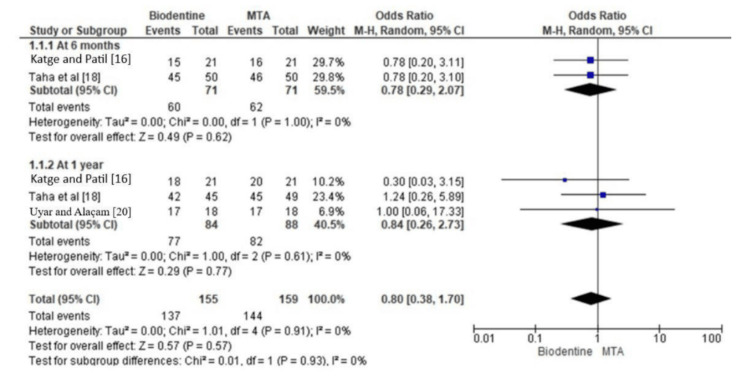
Radiographic success rate of Biodentine as compared to MTA MTA: mineral trioxide aggregate

Figure 4 shows the comparison of radiographic success rate between Biodentine and MTA. The analyses were stratified by follow-up time: six months and one year of follow-up, respectively. In the follow-up of six months, this plot illustrates the estimates of individual studies and their 95% CIs and the summary estimate and its 95% CI. This analysis includes the studies by Katge and Patil [16] and Taha et al. [18]. The summary estimate of the OR is 0.78 (95% CI: 0.29, 2.07), suggesting that Biodentine tends to present a similar radiographic success rate compared to MTA in six months. The difference was not statistically significant. The test for heterogeneity did not detect any significant between-study variance (tau-squared = 0.00, I-squared = 0%).

Individual study estimates and their 95% CIs are plotted along with the summary estimate and 95% CI at one-year follow-up, showing that the summary estimate of the OR is 0.84, with a 95% CI between 0.26 and 2.73. Thus, at one year, Biodentine can be said to hold an equal radiographic success rate with MTA. The 95% CI includes the null value of 1. This suggests that the difference is not statistically significant (p = 0.77). There is no significant heterogeneity between studies, as the tau-squared equals 0.00 and I-square equals 0%. The summary estimate for the overall pool in both follow-up periods yields an OR of 0.80 (95% CI: 0.38, 1.70); thus, Biodentine appeared to have a similar radiographic success rate as MTA, but this was not statistically significant. The test for heterogeneity did not demonstrate significant variability between studies, with a tau-squared of 0.00 and an I-squared of 0%. In addition, the test for subgroup differences reveals that there is no difference between the follow-up times of six months and one year (p = 0.93).

Discussion

Upon a collective comparison of the results of each trial included in our review, it is intriguing to realize similarities and dissimilarities among the general inferences drawn. Gözetici-Çil et al. [15] and Rahman and Goswami [17] are quite similar in that both studies advocate that Biodentine is a potential treatment option for carious lesion management, resulting in very high success rates with very low postoperative pain. This is remarkable because both investigations used Biodentine as a treatment material, although in different indications, namely, SRSD with/without CS and indirect pulp treatment.

In contrast, Katge and Patil [16] and Uesrichai et al [19] have made inferences different from what was mentioned above. Katge and Patil [16], while Uesrichai et al. concluded that Biodentine could serve as an alternative to ProRoot MTA for partial pulpotomy procedures in pediatric patients [19]. What they share in common is that both have evaluated the effectiveness of Biodentine against other materials, one against MTA and the other against ProRoot MTA; however, their results are unique, both in specific context and measured outcomes.

Taha et al. [18] and Uyar and Alaçam [20] had similar results since both studies suggested that Biodentine was an effective material for pulpotomy procedures, having a high success rate and substantial reduction in pain scores. This indeed shows the similarity in the two studies, since in both studies, Biodentine was employed as a treatment material in the context of pulpotomy procedures. However, Uyar and Alaçam also mentioned that there was no significant difference between the three groups [20], which makes their study slightly different from other studies. This may point out that the choice of material should be according to individual clinical circumstances and preference, rather than having one superior material over the others.

Concerning divergences, the studies by Gözetici-Çil et al. [15] and Katge and Patil [16] are outliers among the selected articles, since both have tested the effectiveness of Biodentine in different scenarios, namely, SRSD with/without CS and indirect pulp capping, respectively. The study of Uesrichai et al. is also an outlier because it focused on the efficacy of Biodentine as a substitute for ProRoot MTA in partial pulpotomy of pediatric patients [19].

Calcium hydroxide-based release from calcium silicate cements, with the use of Biodentine, for example, triggers pulp reparative processes [21]. The histological studies demonstrated that the homogeneous dentin bridge appeared at the site of pulp exposure, either after direct or indirect pulp capping with Biodentine. In this way, reparative dentin formation and the antibacterial effects of the cement guarantee the long-term preservation of pulp vitality [22,23]. Although the antibacterial action of cement's alkaline pH may be partly responsible, reparative dentin formation is stimulated by transforming growth factor-ß1 that is released from pulp cells [24,25].

The multifactorial etiology of open apex places trauma as one of the major predisposing factors. It can produce gross alterations in pulpal microcirculation, resulting from irritation of pulp tissue and leading to pulpal necrosis with subsequent arrested root formation [4]. There may also be thermal and chemical injuries sustained by the pulpal tissue, resulting in incomplete root development [26-29]. Iatrogenic root end enlargement is also possible due to inappropriate working length management and further enlargement with hand and rotary files [30]. Besides, dental anomalies include dens evaginatus and dens invaginatus, which are also considered one cause of open apices. A clear understanding of the pathogenesis may provide an appropriate treatment outline for immature necrotic teeth [31,32].

When we compare our results with other reviews carried out across similar objectives as ours [33,34], we noticed quite a few similarities and differences. A similar emphasis on the importance of regenerative dentistry in managing open apices was evident from the most relevant literature [33-37]. Gill et al. referred to the regeneration and revascularization procedures [33], while Saxena et al. discussed the successful newer biomimetic materials such as MTA and biodentine in apexogenesis and apexification procedures, respectively [37]. Our review, therefore, supports the opinion that regenerative dentistry may also present a more conservative method in generating an apical barrier.

Limitations

Both our qualitative and quantitative analyses need to be considered with caution, with the primary limitation being the limited number of trials that we included in the review. Moreover, the lack of significant differences in clinical and radiographic success rates between Biodentine and other materials may be attributed to the variability in study designs, follow-up periods, and outcome measures employed across the included studies. These limitations highlight the need for further research to provide more robust evidence.

Clinical recommendations

Based on our findings, we advocate that clinicians should consider Biodentine as one of the treatment options for apexogenesis in young permanent mandibular molars. We do, however, want to emphasize caution in interpreting these results and individual clinical circumstances and patient preferences in choosing a material for apexogenesis. Lastly, we would like to recommend that investigators give priority to future research regarding overcoming several of the limitations of this study, such as the need for additional high-quality studies with standardized outcome measures and longer follow-up. More so, treatment protocols and materials used should be subject to continuous research due to the intricacy of the procedure and responses from patients when dealing with apexogenesis in young permanent mandibular molars.

## Conclusions

Our assessments indicate that Biodentine might be a promising treatment modality for apexogenesis in young permanent mandibular molars, yielding clinically and radiographically successful rates comparable to those obtained using other calcium compounds and MTA. We do recognize that any conclusions derived from the results of this study should be considered cautiously in light of certain limitations. The results hence indicate that Biodentine may be the alternative; however, much more research needs to be done to understand the efficacy and the best way of using it fully.
